# The *Mycobacterium tuberculosis *Rv2540c DNA sequence encodes a bifunctional chorismate synthase

**DOI:** 10.1186/1471-2091-9-13

**Published:** 2008-04-29

**Authors:** Fernanda Ely, José ES Nunes, Evelyn K Schroeder, Jeverson Frazzon, Mário S Palma, Diógenes S Santos, Luiz A Basso

**Affiliations:** 1 Centro de Pesquisas em Biologia Molecular e Funcional, Pontifícia Universidade Católica do Rio Grande do Sul, RS 90619-900, Porto Alegre, Brazil; 2 Instituto de Ciência e Tecnologia de Alimentos, Universidade Federal do Rio Grande do Sul, RS 91501-970, Porto Alegre, Brazil; 3 Departamento de Biologia/CEIS, Universidade Estadual Paulista, SP 13506-900, Rio Claro, Brazil

## Abstract

**Background:**

The emergence of multi- and extensively-drug resistant *Mycobacterium tuberculosis *strains has created an urgent need for new agents to treat tuberculosis (TB). The enzymes of shikimate pathway are attractive targets to the development of antitubercular agents because it is essential for *M. tuberculosis *and is absent from humans. Chorismate synthase (CS) is the seventh enzyme of this route and catalyzes the NADH- and FMN-dependent synthesis of chorismate, a precursor of aromatic amino acids, naphthoquinones, menaquinones, and mycobactins. Although the *M. tuberculosis *Rv2540c (*aroF*) sequence has been annotated to encode a chorismate synthase, there has been no report on its correct assignment and functional characterization of its protein product.

**Results:**

In the present work, we describe DNA amplification of *aroF*-encoded CS from *M. tuberculosis *(*Mt*CS), molecular cloning, protein expression, and purification to homogeneity. N-terminal amino acid sequencing, mass spectrometry and gel filtration chromatography were employed to determine identity, subunit molecular weight and oligomeric state in solution of homogeneous recombinant *Mt*CS. The bifunctionality of *Mt*CS was determined by measurements of both chorismate synthase and NADH:FMN oxidoreductase activities. The flavin reductase activity was characterized, showing the existence of a complex between FMN_ox _and *Mt*CS. FMN_ox _and NADH equilibrium binding was measured. Primary deuterium, solvent and multiple kinetic isotope effects are described and suggest distinct steps for hydride and proton transfers, with the former being more rate-limiting.

**Conclusion:**

This is the first report showing that a bacterial CS is bifunctional. Primary deuterium kinetic isotope effects show that C_4_-*proS *hydrogen is being transferred during the reduction of FMN_ox _by NADH and that hydride transfer contributes significantly to the rate-limiting step of FMN reduction reaction. Solvent kinetic isotope effects and proton inventory results indicate that proton transfer from solvent partially limits the rate of FMN reduction and that a single proton transfer gives rise to the observed solvent isotope effect. Multiple isotope effects suggest a stepwise mechanism for the reduction of FMN_ox_. The results on enzyme kinetics described here provide evidence for the mode of action of *Mt*CS and should thus pave the way for the rational design of antitubercular agents.

## Background

Tuberculosis (TB) remains a major global health concern. Its causative agent, *Mycobacterium tuberculosis*, has been estimated to infect approximately one-third of the world's population [1], and approximately 30 million people have died from the disease in the past decade [2]. The World Health Organization estimated a total of 9 million new cases of TB and approximately 2 million deaths from this disease in 2004, second only to AIDS among infectious diseases [3]. The emergence of drug resistant isolates of *M. tuberculosis*, particularly of multi drug-resistant TB (MDR-TB), defined as resistant to at least isoniazid and rifampicin [4], imposes a great challenge to public health [5]. Treatment of MDR-TB requires the administration of second-line drugs that are more toxic and less effective [6]. More recently, it was reported cases of extensively drug-resistant (XDR) TB, which are defined as cases in persons with TB whose isolates were resistant to isoniazid and rifampicin and at least three of the six main classes of second-line drugs (aminoglycosides, polypeptides, fluoroquinolones, thioamides, cycloserine, and *para*-aminosalicylic acid) [7]. XDR-TB has a wide geographic distribution and it raises the bleak prospect of a future epidemic of virtually untreatable TB. New antimycobacterial agents are thus needed to improve the treatment of MDR- and XDR-TB, to shorten the treatment course and increase patient compliance, and to provide more effective treatment of latent TB infection.

A valuable approach to the development of selective antimicrobial chemotherapy is to exploit the inhibition of targets unique and vital to the pathogen [8]. The enzymes of the shikimate pathway are attractive examples of these targets because this route is essential in higher plants, fungi, bacteria and algae and is absent in mammals [9,10]. In *M. tuberculosis*, the shikimate pathway leads to the biosynthesis of a wide range of primary and secondary metabolites, including aromatic amino acids, folate, naphthoquinones, menaquinones and mycobactins [11]. Disruption of *aroK*-encoding shikimate kinase gene has recently shown that the shikimate pathway is essential for *M. tuberculosis *viability [12], which establishes the enzymes of this pathway as potential targets for the development of new antimycobacterial agents.

**Figure 1:**
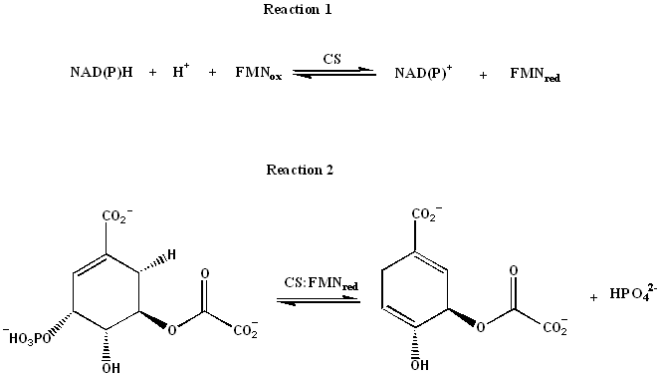
Reactions catalysed by bifunctional CSs.

Homologues to the shikimate pathway enzymes were identified in the complete genome sequence of *M. tuberculosis *H37Rv [13]. Among them, chorismate synthase (CS; EC 4.2.3.5; 5-*O*-(1-carboxyvinyl)-3-phosphoshikimate phosphate lyase) encoding gene (*aroF*, Rv2540c) was proposed to be present by sequence homology. CS catalyzes an unusual 1,4-*anti*-elimination of the 3-phosphate group and the C-(6*pro*R) hydrogen from 5-enolpyruvylshikimate-3-phosphate (EPSP) forming chorismate and phosphate [14]. Although there is no overall change in its redox state, there is an absolute requirement for a reduced flavin mononucleotide (FMN_red_) [15,16]. Another interesting feature of CSs from different organisms is how reduced FMN_red _is obtained, which divides these enzymes into two classes: monofunctional and bifunctional [17]. The CSs from fungi are bifunctional as they display a second enzymatic activity (Figure 1), an NAD(P)H-dependent (dependent of reduced form of nicotinamide adenine dinucleotide) flavin reductase (described as "diaphorase" activity in a number of papers), which confers them an intrinsic ability to reduce flavin using NAD(P)H [18]. The CSs from plants and *Escherichia coli *are monofunctional as they do not possess this activity and are active only in anaerobic conditions in the presence of either chemically or enzymatically reduced flavin [16]. However, there has been no report on both confirmation of the correct assignment of Rv2540c (*aroF*) as the *M. tuberculosis *chorismate synthase (*Mt*CS) coding sequence and functional characterization of its protein product. Moreover, there has been no report on whether *Mt*CS is bifunctional or monofunctional.

Here we report the amplification and cloning of Rv2540c DNA sequence (putative *aroF *gene) from *M. tuberculosis*, and heterologous overexpression of the *Mt*CS protein. The recombinant protein was purified to homogeneity using a three-step purification protocol and its identity was confirmed by N-terminal sequencing, and electrospray ionization mass spectrometry (ESI-MS). The oligomeric state of *Mt*CS was determined by gel filtration. We also show that CS from *M. tuberculosis *is bifunctional, as in fungi, based on activity measurements of both chorismate synthase and NADH-dependent flavin reductase activities. Moreover, FMN appears to be bound to *Mt*CS based on ionic exchange chromatography, and FMN_ox _(oxidized flavin mononucleotide) and NADH binding to *Mt*CS were assessed by spectroscopic measurements at equilibrium. The apparent kinetic constants for the holoenzyme *Mt*CS-FMN_ox _for NADH were determined. The NADH-dependent flavin reductase activity was characterized by isotope effects. Measurements of primary deuterium and solvent kinetic isotope effects were carried out to probe the nature of the rate-limiting step of the redox reaction. Multiple isotope effects were also determined showing that a stepwise mechanism is involved in this reaction. Proton inventory on the maximal velocity allowed us to address the number of kinetically important transferred protons. The results presented here are important to understand the reaction mechanism of this enzyme, which should pave the way for the rational-based design of *Mt*CS inhibitors with potential antitubercular activity and low toxicity.

## Results and Discussion

### Molecular cloning, expression, and purification of *Mt*CS

**Figure 2:**
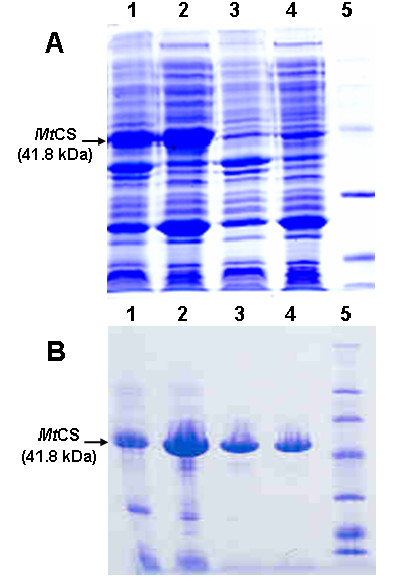
**Recombinant *Mt*CS protein expression and purification**. **(A) **Analysis of *Mt*CS expression using *E. coli *Rosetta(DE3) host cells by SDS-poliacrilamide gel 12%. Lanes: 1 – pellet Rosetta(DE3)+pET23a(+)::*aroF*; 2 – soluble extract Rosetta(DE3)+pET23a(+)::*aroF; *3 – pellet Rosetta(DE3)+pET23a(+) (negative control); 4 – soluble extract Rosetta(DE3)+pET23a(+) (negative control); 5 – protein molecular weight standards, high range (Gibco) (43 kDa; 29 kDa; 18 kDa; 14 kDa) **(B) **Analysis of purification steps of *Mt*CS by SDS-polyacrylamide gel 12%. Lanes: 1 – soluble extract after dialysis; 2 – soluble extract after Q Sepharose Fast Flow column; 3 – CS after Phenyl-Sepharose column; 4 – CS after MonoQ column; 5 – protein molecular weight standards, high range (Gibco) (200 kDa; 97 kDa; 68 kDa; 43 kDa; 29 kDa; 18 kDa; 14 kDa).

PCR amplification of Rv2540c DNA sequence (putative *aroF *gene) from genomic *M. tuberculosis *DNA yielded a fragment with the expected length (1206 bp). The fragment was cloned into pET23a(+) expression vector, and the *aroF *gene was sequenced, which confirmed its identity and the absence of PCR (Polymerase Chain Reaction)-introduced mutations. A number of *E. coli *host strains were employed to express *Mt*CS, including BL21(DE3), Origami(DE3), and BL21*trxB*(DE3), but no expression could be obtained. Different cultivating temperatures (25°, 30° and 37°C) and presence or absence of isopropyl β-D-thiogalactoside (IPTG) were also employed to no avail (data not shown). *Mt*CS overexpression could only be achieved in *E. coli *Rosetta(DE3) host cells, which provide tRNAs for codons that are rarely used by *E. coli*. In agreement, ten rare codons were identified on *M. tuberculosis aroF *sequence (7 × CCC, AUA, CUA, GGA). Sodium dodecyl sulfate polyacrylamide gel electrophoresis (SDS-PAGE) analysis revealed a cell extract containing a significant amount of a soluble protein with an apparent molecular weight in agreement with the predicted value based on amino acid sequence for *Mt*CS (41.8 kDa) (Figure 2A).

Interestingly, recombinant protein expression was achieved in *E. coli *Rosetta(DE3) host cells grown for 24 h at 37°C in the absence of IPTG induction. It has been previously shown that the *lac*-controlled systems, including the pET system, could have a high-level protein expression, in the absence of inducer. This phenomenon occurs in the cell stationary phase, when there is the presence of a complex medium, cyclic AMP, acetate and low pH [19]. Recently, another shikimate pathway enzyme from *M. tuberculosis*, 3-Deoxy-D-*arabino*-heptulosonate 7-phosphate (DAHP) synthase, was reported to be expressed only in the absence of IPTG [20]. The three-step purification protocol yielded approximately 55 mg of *Mt*CS from 6 liters of cell culture. No other protein can be observed on SDS-PAGE, which indicates the high level of *Mt*CS purity (Figure 2B). The *Mt*CS activity before the purification can hardly be measured, probably due to interfering compounds, and thus only the amount of total recombinant protein could be assessed. The ammonium sulfate precipitation of purified *Mt*CS resulted in total inactivation of the enzyme (data not shown). Homogeneous recombinant protein was thus instantaneously frozen in liquid nitrogen in the absence of ammonium sulfate, which proved to be a convenient protocol for active homogeneous recombinant *Mt*CS protein storage.

### Analysis of purified *Mt*CS

The ESI-MS of homogeneous *Mt*CS revealed a subunit molecular mass of 41,804 Da, consistent with the theoretical molecular mass (41,792 Da). The first 16 N-terminal amino acid residues were identified as MLRWITAGESHGRALV, which confirmed the identity of homogeneous *Mt*CS and presence of N-terminal methionine residue. Analytical gel filtration chromatography revealed a single peak of approximately 104 kDa, suggesting that *Mt*CS is a dimer in solution, which is in agreement with the hydrodynamic properties of the recombinant protein assessed by sedimentation velocity and sedimentation equilibrium [18].

### The CS activity of *Mt*CS

The synthesis of EPSP was carried out in a vial containing the enzymes *Mt*SK and *Mt*EPSPS, which convert shikimate, ATP and phosphoenolpyruvate (PEP) to 5-enolpyruvylshikimate-3-phosphate (EPSP), ADP and Pi. The equilibrium of the forward reaction was displaced using Purine Nucleoside Phosphorylase (PNP), which consumes Pi, increasing the final concentration of EPSP in the reaction mixture. The enzymes were removed by ultrafiltration to avoid any residual *Mt*EPSPS enzyme activity that could release Pi in solution, which would thus interfere with specific measurements of *Mt*CS enzyme activity. Conversion of EPSP to chorismate and Pi catalyzed by *Mt*CS enzyme activity (Figure 1) was determined by measuring the release of Pi using PNP, which converts 2-amino-6-mercapto-7-methylpurine ribonucleoside (MESG) and Pi to ribose-1-phosphate and 7-methyl-6-thio-guanine base, monitoring absorbance of the latter at 360 nm. The enzymatic activity of homogenous *Mt*CS was dependent on enzyme volume added to the reaction mixture (data not shown), showing that the initial velocity is proportional to total enzyme concentration and that true initial velocities are being measured. The conversion of EPSP to chorismate in aerobic conditions by the addition of FMN_ox _and NADH is the first solid evidence that *Mt*CS is bifunctional, since the monofunctional CSs can only be assayed under strictly anaerobic conditions in the presence of chemically or enzymatically reduced flavin [16]. The specific activity of bifunctional *Mt*CS (0.004 U.mg^-1^) is approximately 175-fold lower than the specific activity of bifunctional *Neurospora crassa *CS (*Nc*CS; 0.7 U.mg^-1^) [21]. In the presence of oxygen the CS activity is limited by the reoxidation of FMN_red _cofactor; but in bifunctional CSs, NAD(P)H is used in consecutive cycles to maintain the FMN in its reduced form (Figure 1). Moreover, the structures of CS complexed with FMN from *Helicobacter pylori *[22] and *Streptococcus pneumoniae *[23] have shown that there are a number of positively charged amino acids in the FMN binding pocket, which could increase the reduction potential of FMN_ox_/FMN_red _couple, likely permitting the decrease of the rate of reoxidation. Indeed, in bifunctional *Nc*CS the redox potential of the couple FMN_red_/FMN_ox _was determined to be -167 mV, *i.e*. 40 mV more positive than that of the free couple in solution (-207 mV) [24].

### The NADH:FMN-oxidoreductase activity of *Mt*CS

**Figure 3:**
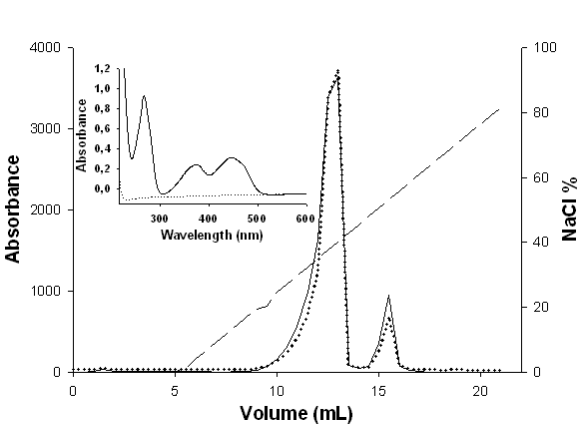
**Elution profile of incubated *Mt*CS and FMNox, and UV-Vis spectrum showing stable binary complex formation**. Elution of *Mt*CS and FMN_ox_incubation in a HiTrapQ HP column upon a linear gradient from 0 to 1 M NaCl (dashed line). The absorbance was monitored at 445 nm (dotted line) and 280 nm (solid line). The first peak corresponds to free FMN_ox_, and the second peak corresponds to *Mt*CS-FMN_ox _complex. Inset: UV-Vis spectrum ranging from 210 nm to 600 nm of the second pool (solid line) and Tris-HCl (dotted line). All characteristic peaks of FMN_ox _can be observed (267 nm, 373 nm, and 445 nm).

The NADH-dependent reduction of FMN_ox _was measured in the forward reaction monitoring the decrease in NADH concentration. The NADH:FMN-oxidoreductase activity of homogenous *Mt*CS is dependent on the enzyme volume added to the reaction mixture; therefore the initial velocity is proportional to total enzyme concentration, and true initial velocities are being measured. To verify the stability of the *Mt*CS-FMN_ox _complex, *Mt*CS was incubated with excess FMN_ox _and loaded on an anion exchange column. Two peaks were observed and collected into separated fractions measuring absorbance at 280 nm and 445 nm, and pooled (Figure 3). To roughly estimate the presence of protein in the two pools, coomassie-blue was added to them and only the second pool was shown to contain protein (indicating presence of recombinant *Mt*CS), while the first was not stained (indicating absence of recombinant *Mt*CS). The UV-Vis spectrum for the second pool containing recombinant *Mt*CS protein showed that all the characteristic absorbance peaks of FMN_ox _(267, 373, and 445 nm) could be observed (Figure 3 inset). These results suggest that *Mt*CS and FMN_ox _form a stable binary complex.

Although the FMN_ox _usually appears as a prosthetic group in NAD(P)H:FMN oxidoreductases from other organisms [25], it was not clear whether in *Mt*CS the FMN is covalently bound. CSs from other organisms, such as that from *E. coli*, showed low affinity for FMN_ox_; but the value of Kd for FMN_ox _depends strongly on EPSP binding, decreasing ca. 1000-fold in the presence of this substrate (from 30 μM to 20 nM) [26]. On the other hand, EPSP has a much smaller effect on the affinity of bifunctional *Nc*CS to FMN_ox _[21]. Interestingly, the purified *Mt*CS is FMN-free, which could be an effect of the insufficient intracellular concentration of FMN, probably due to *Mt*CS overexpression into the host cell. As described below, FMN appears to have a value lower than 20 μM for the overall dissociation constant for *Mt*CS-FMN binary complex formation at equilibrium. Accordingly, it appears to be more likely that FMN is lost during the protein purification protocol because of a rather weak affinity of *Mt*CS for FMN. Accordingly, the kinetic data were collected considering the holoenzyme as the *Mt*CS-FMN_ox _complex brought about by incubating *Mt*CS and near-saturating FMN_ox _concentration.

**Figure 4:**
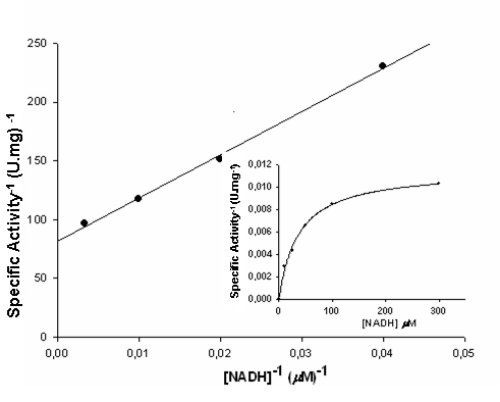
**Determination of steady-state kinetic parameters**. Double-reciprocal plot of initial velocity data for holoenzyme *Mt*CS-FMN_ox _in the presence of varying concentration of NADH (10, 25, 50, 75, 100, 200, 300 μM). Inset: Michaelis-Menten representation. The data were fitted to equation 1, yielding the following values: *K*_NADH _= 36 ± 4 μM, *k*_cat _= 8.3 (± 0.3) × 10^-3 ^s^-1 ^and *k*_cat_/*K*_NADH _= 231 M^-1 ^s^-1^.

The oxidoreductase activity is functionally independent of CS activity, since the consumption of NADH occurs in the absence of EPSP. Considering the low specific activity of NADH:FMN-oxidoreductase in *Mt*CS, all measurements were made in triplicate, including the controls. Accordingly, the apparent kinetic parameters for the oxidoreductase activity were determined in the absence of EPSP in steady-state kinetic experiments. No activity was observed when NADPH was used at concentrations ranging from 100 to 500 μM (data not shown). Kinetics of reduction of the *Mt*CS-bound FMN_ox _by NADH was determined using fixed amount of holoenzyme and varying levels of NADH (Figure 4). The data were fitted to equation 1, and yielded the following values: *K*_NADH _= 36 ± 4 μM, *k*_cat _= 8.3 (± 0.3) × 10^-3 ^s^-1 ^and *k*_cat_/*K*_NADH _= 231 M^-1 ^s^-1^. The *K*_NADH _observed for *Mt*CS is similar to the *K*_NADPH _determined for *Nc*CS [24] and *Sc*CS [27] (43 μM and 70 μM, respectively). In other NAD(P)H:FMN oxidoreductases (EC 1.5.1.30), which are present in various organisms, the apparent kinetic constants show significant variation. For instance, the *K*_NAD(P)H _can vary from 208 μM in *Rhodococcus erythropolis *[28] to 0.85 μM in *Bacillus subtilis *[29].

### Equilibrium measurements of FMN_ox _and NADH binding to *Mt*CS

**Figure 5:**
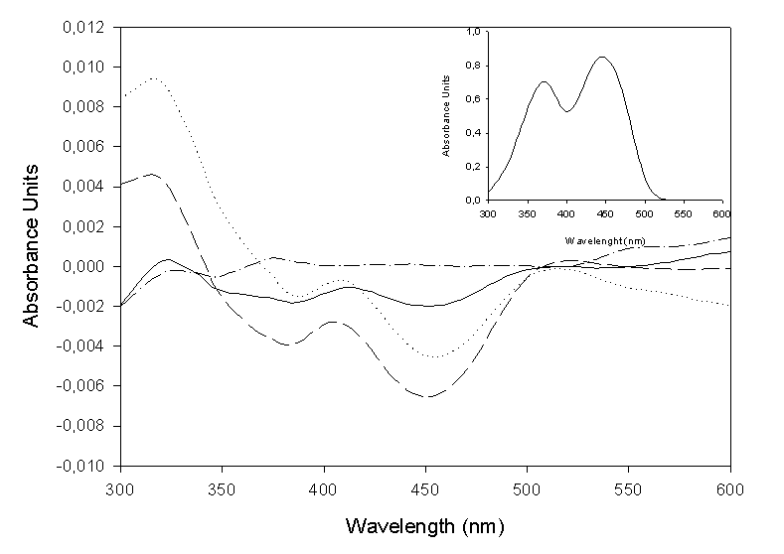
**UV-visible difference spectroscopy**. Difference absorbance spectra were recorded from 300 to 600 nm. The difference spectra are as follows: dash-dotted line (-·-), 35 μM *Mt*CS; solid line (--), 35 μM *Mt*CS in the presence of 10 μM FMN_ox_; dashed line (- -), 35 μM *Mt*CS in the presence of 20 μM FMN_ox_; and dotted line (·····), 35 μM *Mt*CS in the presence of 30 μM FMN_ox_. The inset shows absorbance spectrum of 100 μM free FMN_ox_.

The binding of FMN_ox _to *Mt*CS was assessed by UV-visible difference spectra as described by Kitzing et al. [21]. The observed spectral changes are characterized by hypochromic effects on the flavin absorbance at both 378 nm and 450 nm up to 20 μM FMN_ox _(Figure 5). At FMN_ox _concentrations larger than 30 μM there is a hyperchromic effect that is probably due to free oxidized FMN in solution (Figure 5 – inset). The spectral changes at low FMN_ox _concentrations yielded low values for the difference spectra and thus no reliable data could be obtained at concentrations lower than 10 μM. Accordingly, the data presented here allow only to estimate an upper limit value of 20 μM for the FMN_ox _equilibrium dissociation constant. We have also tried to determine the dissociation constant of FMN_ox _from protein fluorescence titration as recently described by Broco et al [30], which could allow reliable measurement of oxidized FMN binding to *Mt*CS at low concentrations of FMN_ox_. However, there was no quench/enhancement in protein fluorescence upon FMN_ox _binding to *Mt*CS (data not shown). At any rate, the UV-visible difference spectra show *Mt*CS-FMN_ox _binary complex formation.

**Figure 6:**
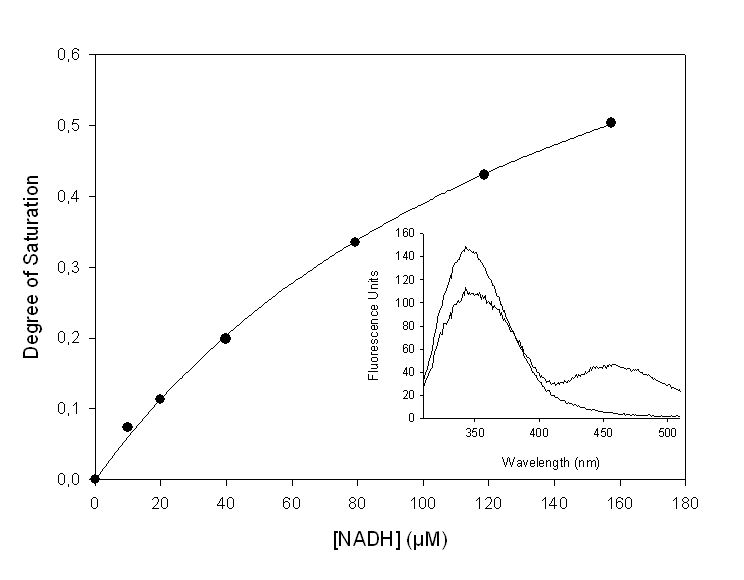
**Equilibrium binding of NADH to *Mt*CS assessed by monitoring the protein fluorescence quench upon binary complex formation**. The binding of NADH to *Mt*CS causes a quench in protein fluorescence (λ_exc _= 290 nm; 310 ≤ λ_em _≤ 510 nm; with a maximum at 345.5 nm). The *Mt*CS enzyme solution (1 μM) was titrated with increasing concentrations of NADH, and the data points (fluorescence intensities at 345.5 nm) were fitted to a hyperbolic equation (solid line). Inset: Emission spectra of free *Mt*CS (1 μM) and enzyme in the presence of 80 μM NADH. Emission spectrum of free enzyme shows a peak at 345.5 nm and no emission at ~450 nm. Emission spectrum of *Mt*CS in the presence of NADH shows a quench in protein fluorescence concomitant to an increase in nucleotide fluorescence at ~450 nm.

The equilibrium binding of NADH to *Mt*CS was assessed by fluorescence spectroscopy. There is a quench in protein fluorescence upon NADH binding to *Mt*CS (Figure 6 – inset). A titration curve showing the quench in protein fluorescence upon the binding of NADH to *Mt*CS is given in Figure 6. The data were fitted to a hyperbolic equation yielding a dissociation constant value of 156 ± 12 μM. Even though the NADH-binding site remains unknown, the results presented here clearly indicate that there is indeed an NADH binding site on *Mt*CS enzyme.

### Primary deuterium kinetic isotope effects

**Figure 7:**
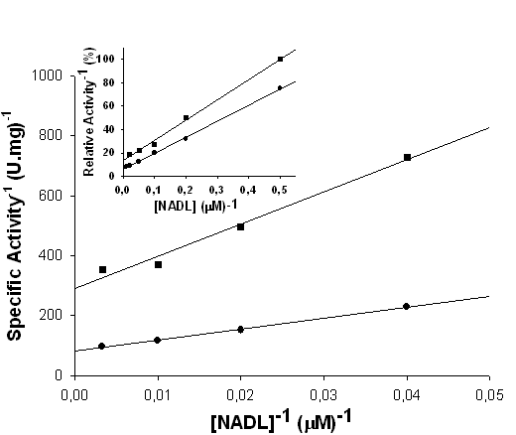
**Primary and multiple deuterium kinetic isotope effects**. Measurements of primary deuterium kinetic isotope effects for *Mt*CS yielded values of 3.5 ± 0.2 and 3.0 ± 0.4 for, respectively, ^D^*V *and ^D^*V/K*. Inset: Measurements of primary isotope effect in 90 atom % D_2_O (multiple) yielded values of 1.3 ± 0.1 and 2.2 ± 0.1 for ^D2O^*V*_[4*S*-2H]NADH _and ^D2O^*V/K*_[4S-2H]NADH_, respectively. In both [4*S*-^1^H]NADH (●) or [4*S*-^2^H]NADH (■) is varied in the reaction mixture. L represents either [4*S*-^1^H]NADH or [4*S*-^2^H]NADH.

To probe for rate-limiting steps and determine the stereospecificity of hydride transfer, primary deuterium kinetic isotope effects were determined. Initial velocity data were collected using either [4*S*-^1^H]NADH or [4*S*-^2^H]NADH (Figure 7), and values of 3.5 ± 0.2 and 3.0 ± 0.4 were determined for, respectively, ^D^*V *and ^D^*V/K*. The magnitude of the primary isotope effects when the [4*S*-^2^H]NADH substrate is used indicates that C_4_-*proS *hydrogen is being transferred during the reduction of FMN_ox _catalyzed by *Mt*CS. Isotope effects on *V *are related to events following the formation of the complex capable of undergoing catalysis (*Mt*CS-FMN_ox_-NADH in the case studied here), including chemical steps, possible enzyme conformational changes, and product release. Isotope effects on *V/K *report on steps in the reaction mechanism from the binding of the labeled substrate to the first irreversible step, usually product release [31]. The values for primary deuterium isotope effects of biochemical interest usually range from 3 to 7 [32], and steps such as conformational changes accompanying hydride transfer and product release may account for the lower values. In particular, primary deuterium kinetic isotope effects typically range from 1 to 3 for enzyme reactions involving NAD(P)H oxidation [33]. The ^D^*V *value obtained for *Mt*CS-FMN_ox _(3.5) suggests that the hydride transfer contributes significantly to the rate-limiting step of the FMN reduction reaction. The value of 3.0 for the ^D^*V/K *indicates that NADH is not sticky, since ^D^*V/K *values for sticky substrates are around unity [34]. It should be remembered that a substrate is sticky if it reacts to give products as fast as, or faster than, it dissociates from the enzyme. The stickiness of a substrate depends on the external part of its commitment: the larger the external commitment, the sticker the substrate [34].

### Solvent isotope effects and proton inventory

**Figure 8:**
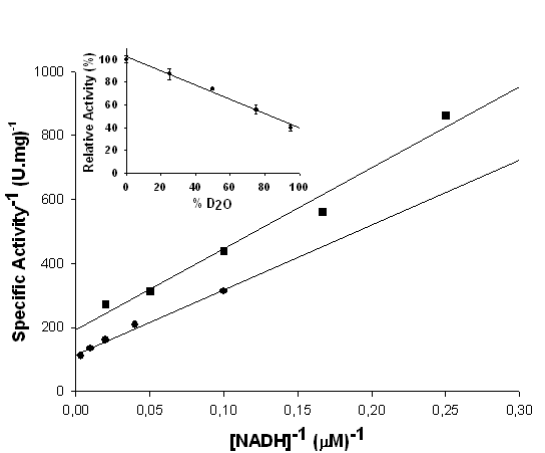
**Solvent deuterium kinetic isotope effects and proton inventory**. Solvent isotope effects for *Mt*CS. NADH is varied in the reaction mixture containing either 0 (●) or 91 (■) atom % D_2_O. Values of 1.7 ± 0.3 and 1.3 ± 0.1 were obtained for ^D2O^*V *and ^D2O^*V/K*, respectively. Inset: Proton inventory at saturating concentration of NADH, indicating that a single proton transfer from solvent contribute to the rate of flavin reduction.

Solvent isotope effects were employed to assess the contribution of solvent proton transfer to the rate of flavin reduction. Initial velocity data were collected in either H_2_O or 91 atom % D_2_O (Figure 8), and values of 1.7 ± 0.3 and 1.3 ± 0.1 were obtained for ^D2O^*V *and ^D2O^*V/K*, respectively. The magnitude of the solvent isotope effects indicate that proton transfer partially limit the rate of FMN reduction reaction. In order to determine the number of kinetically important protons transferred during the reaction, a proton inventory on *V *was carried out. The mole fraction of D_2_O was varied from 0 to 80%, and a linear relationship between *V *and the mole fraction of D_2_O was observed (Figure 8, inset). This result indicates that a single proton transfer gives rise to the observed solvent isotope effect [35].

### Multiple isotope effects

Significantly different magnitudes between primary and solvent kinetic isotope effects may indicate that they are reporting on distinct steps of a reaction, as reported for the NADPH-dependent ketoacyl-ACP reductase from *S. pneumoniae *[36]. The primary isotope effect values (^D^*V *= 3.5 and ^D^*V/K *= 3.0) are larger than those for solvent isotope effects (^D2O^*V *= 1.7 and ^D2O^*V/K *= 1.3), suggesting that proton and hydride transfer may take place in two distinct transition-states, with the latter being more rate-limiting for the *Mt*CS-catalyzed reaction. Multiple isotope effects studies are able to distinguish whether two different isotopic substitutions affect the same or different chemical steps. Theory predicts that if protonation and hydride transfer occur in the same transition state, the primary isotope effects will be larger or unchanged with D_2_O as compared to H_2_O. On the other hand, if hydride transfer and protonation occur in distinct steps, the primary isotope effects will be smaller with D_2_O as solvent, as proton transfer will become more rate limiting [37,38]. Multiple isotope effects were thus evaluated to distinguish whether two different isotopic substitutions affect the same or different chemical steps, *i.e*., if the reduction of FMN_ox _catalyzed by *Mt*CS occurs, respectively, in a concerted or in a stepwise mechanism. Initial velocity data were collected and values of 1.3 ± 0.1 and 2.2 ± 0.1 were obtained for ^D2O^*V*_[4*S*-2H]NADH _and ^D2O^*V/K*_[4*S*-2H]NADH_, respectively (Figure 7, inset). The values for the primary kinetic isotope effects on *V *and *V/K *in D_2_O were smaller than the effects in H_2_O, thereby suggesting a stepwise mechanism for the reduction of FMN_ox_. A mechanism for bifunctional *Nc*CS has been proposed and involves an electron transfer from FMN_red _to C1 EPSP and C-O bond cleavage, protonation of the leaving phosphate group by His17, tautomerization of the resulting C4(a)-neutral flavin semiquinone to a radical species with concomitant abstraction of the C-(6*pro*R) hydrogen of the dephosphorylated substrate intermediate, and reduced flavin deprotonation restores the initial state of the cofactor [24]. More recently, it has been shown for *Nc*CS that the carboxylate group of Asp367 participates in the water-mediated deprotonation of the N(5) atom of the isoalloxazine ring system of FMN_red _that is involved in abstraction of C-(6*pro*R) hydrogen of EPSP [39]. A comparison of the primary sequences of CSs from different sources has shown that these residues (His11 and Asp343, *M. tuberculosis *numbering) are conserved [18]. Interestingly, His11 and Asp343 are present in monofunctinal and bifunctional CSs and across all known species [40], and these residues are part of the characteristic CS signature sequence [23]. The role of His11 and Asp343 in the mode of action of bifunctional *Mt*CS should await site-directed mutagenesis studies that are currently underway in our laboratory.

## Conclusion

It has recently been pointed out that we are currently unable to predict bifuncionality based on sequence information and three-dimensional structures available at present because of difficulties in identifying the NAD(P)H-binding site of bifunctional CSs [40]. Incidentally, the three-dimensional structural model [41] and X-ray diffraction structure [18] of *Mt*CS employed the crystal structure of CS from *Streptococcus pneumoniae *[23] as template, which is a monofunctional CS. It is thus necessary to measure CS enzyme activity to show whether it depends on a source of free reduced FMN (monofunctional) or can directly reduce FMN cofactor at the expense of NAD(P)H (bifunctional). Here we present experimental evidence that recombinant *Mt*CS is bifunctional. To the best of our knowledge, this is the first report showing that a bacterial CS is bifunctional. We also show that FMN and NADH bind to free *Mt*CS. Primary deuterium kinetic isotope effects of the NADH-dependent flavin reductase activity of *Mt*CS showed that C_4_-*proS *hydrogen is transferred during the reduction of FMN_ox_, and that hydride transfer contributes significantly to the rate-limiting step of FMN reduction. Solvent deuterium kinetic isotope effects suggest that proton transfer from solvent partially limits the rate of FMN reduction and that a single proton is transferred from solvent. Multiple isotope effects indicate that a stepwise mechanism for the reduction of FMN_ox_. Expression of functional proteins in soluble form has been identified as an important bottleneck in efforts to determine biological activity and crystal structure of *M. tuberculosis *proteins [42]. Moreover, protein purification has become an important asset in any structural genomic effort as there has been increasing demand for homogeneous proteins [43]. Accordingly, the work presented here should contribute to overcome these obstacles for, at least, *Mt*CS and allow functional and structural efforts to be pursued. We provide experimental evidence for the correct assignment of the Rv2540c DNA sequence as a CS-coding *aroF *gene in *M. tuberculosis*, which is a pivotal step for the rational design of inhibitors of *Mt*CS enzyme activity with potential antitubercular action. The availability of *Mt*CS and the experimental evidence given here for an NADH-binding site warrant further efforts to obtain the crystal structure of the binary complex to try to elucidate the structural features of this interaction and the mechanism of action for bifunctional CSs. The results reported here should thus pave the way for further functional and structural studies to guide the rational design of antitubercular agents.

## Methods

### Chemical, reagents, enzymes and bacterial strains

*Pfu *DNA polymerase was from Stratagene. The pET23a(+) expression vector and *E. coli *Rosetta(DE3) host cell were from Novagen. All chromatographic supports, including the molecular weight calibration kits, were purchased from GE Healthcare. The protease inhibitor cocktail was from Roche. Purine nucleotide phosphorylase (PNP, EC 2.4.2.1) and 2-amino-6-mercapto-7-methylpurine ribonucleoside (MESG) were purchased from Molecular Probes. NADH, NAD^+^, oxidized FMN (FMN_ox_), ATP, phosphoenolpyruvate (PEP), *Leuconostoc mesenteroides *glucose-6-phosphate dehydrogenase (type XXIII) and yeast hexokinase (type C-300) were from Sigma Chemical Co. D-glucose-1-d (97 atom % D) was from Aldrich and deuterium oxide (99.9 atom % D) was from Cambridge Isotope Laboratories.

### Molecular cloning, and overexpression of *M. tuberculosis *Rv2540c (*aroF*) DNA sequence

Synthetic oligonucleotide primers complementary to amino-terminal coding and carboxy-terminal noncoding strands of *aroF *(Rv2540c) gene (5' ggt**catatg**ttgcgctggatcaccgcgg 3' and 5' c**ggatcc**tcaaccggagacccgcgcggc 3', respectively) were designed based on the complete genome sequence of M. tuberculosis [13]. These primers, containing 5'*Nde*I and 3'*Bam*HI restriction sites, in bold, were used to amplify *aroF*-CS encoding gene (1,206 bp) from *M. tuberculosis *genomic DNA, using *Pfu *DNA polymerase and standard PCR conditions. The amplified fragment was purified by low melting agarose electrophoresis, digested with *Nde*I and *Bam*HI, and cloned into pET23a(+) expression vector previously digested with the same restriction enzymes. The *aroF *gene identity and the absence of PCR-introduced mutations were confirmed by DNA sequencing. The pET23a *(+)::aroF *was transformed by electroporation into *E. coli *Rosetta(DE3) host cells and selected on LB agar plates containing 50 μg.mL^-1 ^carbenicillin and 34 μg.mL^-1 ^chloramphenicol. Single colonies were used to inoculate 1 L of Luria-Bertani medium containing the same antibiotics and grown for 24 h at 37°C at 180 rpm with no isopropyl β-D-thiogalactoside (IPTG) induction. The cells (4.5 g) were harvested by centrifugation at 3,000 *g *for 30 min at 4°C.

### Recombinant *Mt*CS protein purification

All purification procedures were performed at 4°C. Cells (25 g) were suspended in 75 mL of 50 mM Tris-HCl, pH 7.8 (buffer A) containing proteinase inhibitor cocktail and 0.2 mg.mL^-1 ^lisozyme; and the mixture was stirred for 30 min. The cells were disrupted by sonication and centrifuged at 48,000 *g *for 30 min to remove cell debris. Streptomycin sulfate was added to the supernantant to a final concentration of 1% (w/v) and the mixture was stirred for 30 min. The soluble fraction was collected by centrifugation at 48,000 *g *for 30 min and dialyzed 3 times against 2 L of buffer A. This sample was clarified by centrifugation (48,000 *g *for 30 min), loaded on a Q-Sepharose Fast Flow (2.6 cm × 8.2 cm) anion exchange column previously equilibrated with buffer A and the proteins eluted using a linear gradient from 0.0 to 0.5 M NaCl. The fractions containing *Mt*CS were pooled and ammonium sulfate was added to a final concentration of 0.8 M. This sample was loaded on a High Load Phenyl-Sepharose (1.6 cm × 10 cm) hydrophobic interaction column pre-equilibrated with 50 mM Tris-HCl, pH 7.8, 0.8 M (NH_4_)_2_SO_4_(buffer B). The proteins were fractionated using a linear gradient from 0.8 to 0.0 M (NH_4_)_2_SO_4_. The active fractions were pooled and dialyzed 3 times against 2 L of buffer A. The sample was loaded on a MonoQ (1.6 cm × 10 cm) anion exchange column previously equilibrated with buffer A, and the proteins eluted using a linear gradient of NaCl (0.0 – 0.5 M). The fractions containing homogeneous *Mt*CS were pooled, quickly frozen in liquid nitrogen and stored at -80°C. Samples of each purification step were analyzed by SDS-PAGE [44]. Protein concentration was determined by Bradford method [45], using Bio-Rad protein assay kit (Bio-Rad) and bovine serum albumin as standard.

### Mass spectrometry analysis and N-terminal amino acid sequencing

Recombinant *Mt*CS was analyzed using mass spectrometry in an adaptation of Chassaigne and Lobinski systems [46]. Samples were analyzed on a triple quadrupole mass spectrometer, model QUATRO II, equipped with a standard electrospray (ESI) probe (Micromass, Altrinchan) adjusted to ca. 250 μL.min^-1^. The source temperature (80°C) and needle voltage (3.6 kV) were maintained constant throughout the data collection, applying a drying gas flow (nitrogen) of 200 L.h^-1 ^and a nebulizer gas flow of 20 L.h^-1^. The equipment was calibrated with intact horse heart myoglobin. Approximately 50 pmol of sample was injected into electrospray transport solvent to determine the subunit molecular mass of *Mt*CS. The N-terminal amino acid residues of purified recombinant *Mt*CS were identified by automated Edman degradation sequencing using a PPSQ 21A gas-phase sequencer (Shimadzu).

### Determination of native *Mt*CS molecular mass

The molecular mass of native *Mt*CS was determined by gel filtration using Superdex S-200 (10 mm × 30 cm) column eluted with buffer A containing 200 μM NaCl at 0.4 mL.min^-1^. The protein elution was monitored at 280 nm. The protein molecular weight standards were from Low Molecular Weight and High Molecular Weight Calibration kits (GE Healthcare).

### EPSP synthesis

Since there is no ESPS commercially available, EPSP was synthesized using the enzymes shikimate kinase (*Mt*SK) and EPSP synthase (*Mt*EPSPS) from *M. tuberculosis *[47,48], and PNP [49,50]. The synthesis was carried out using 9.6 mM shikimate, 2.4 mM ATP, 3 mM PEP, 0.4 mM MESG. This reaction mixture was pre-incubated in 50 mM Tris-HCl, 2.5 mM MgCl_2_, 2.5 mM KCl pH 7.6 for 3 min at 25°C. Then the three enzymes were added (2.2 U *Mt*SK, 0.7 U *Mt*EPSPS, 2 U PNP) and the reaction mixture was incubated further at 25°C for 30 min. The enzymes were then removed by ultrafiltration using a Centricon 3000 Da cut-off (Amicon). The filtrated solution, containing the substrate EPSP, was collected and used to measure chorismate synthase activity. The reaction catalyzed by EPSP synthase releases an inorganic phosphate molecule, which is consumed by PNP. This coupled reaction changes the equilibrium constant of ESPS synthase reaction, allowing synthesis of higher concentration of EPSP. One unit (U) of enzyme activity for all enzymes cited in this work is defined as the amount of enzyme that catalyzes the conversion of 1 μmol of substrate per minute at 25°C in an optical path of 1 cm.

### *Mt*CS enzymatic assay for CS activity

The *Mt*CS assay was performed in the forward direction using 15 μL of ESPS-containing solution (see above), 0.04 mM FMN_ox_, 0.3 mM NADH, 0.2 mM MESG, 1 U PNP in buffer A. The reaction mixture was incubated for 4 min at 25°C for total consumption of Pi by PNP, and *Mt*CS was then added. The CS reaction was monitored using a coupled reaction with the PNP enzyme [50]. This enzyme catalyses the cleavage of MESG by the inorganic phosphate molecule which is released by *Mt*CS, and formation of 2-amine-6-mercapto-7-methylpurine is monitored at 360 nm (ε = 11.0 × 10^3^M^-1 ^cm^-1^). The assay was performed at 25°C and monitored using a Shimadzu UV-2550 spectrophotometer. All measurements, including the blanks, were carried out in triplicate to ensure that reliable data were being collected.

### *Mt*CS and FMN_ox _interaction

The stability of the interaction between FMN_ox _and *Mt*CS was tested by incubating *Mt*CS with excess FMN_ox _in buffer A for 12 hours at 4°C in the dark. The mixture was injected into a HiTrapQ HP anion exchange column previously equilibrated with buffer A. The sample was eluted with a linear gradient from 0.0 to 1 M NaCl. Absorption spectrum of the pool of fractions containing FMN_ox _and *Mt*CS was measured at wavelengths values ranging from 210 to 600 nm by a Shimadzu UV-2550 spectrophotometer.

### FMN_ox _and NADH binding to *Mt*CS measured by, respectively, spectrophotometry and spectrofluorimetry

The binding of FMN_ox _to *Mt*CS (35 μM) was assessed as described by Kitzing et al. [21]. UV-visible difference absorbance spectra were monitored at 25°C in a double beam spectrophotometer UV-2550 (Shimadzu) with photometric accuracy of ± 0.002 absorbance units in the range 0 – 0.5 absorbance. The difference spectra were recorded using quartz cells with two chambers (Hellma GmbH & Co) placing identical arrangements in both the sample (sample cuvette) and reference beam (reference cuvette). Spectra were recorded from 300 to 600 nm. Differences between solutions were minimized by filling both sides (sample and reference cuvettes) from single stock solutions of FMN_ox_. Microliter additions of FMN_ox _to both one chamber of the reference cuvette and to the sample cuvette, followed by mixing of enzyme and FMN_ox _solutions for the latter, allowed direct measurements of the difference spectra. This procedure is almost mandatory when change in absorbance signal is relatively small on interaction. All solutions were in 50 mM Tris-HCl, pH 7.8 (buffer A).

Fluorescence titration was performed at equilibrium in a RF-5301PC Spectrofluorophotometer (Shimadzu) at 25°C by making microliter additions of NADH stock solution (10 mM) to 2 mL of 1 μM *Mt*CS (active site concentration) in 50 mM Tris-HCl, pH 7.8 (buffer A). Controls were determined, following exactly the same procedure, by microliter additions of buffer A to account for changes in protein fluorescence due to dilution. In order to assess the NADH inner-filter effect in the fluorimeter, two cuvettes were placed in series so that the contents of the first cuvette acted as a filter of the excitation light and the light emitted from the second cuvette detected. To the first cuvette, NADH was added, while the second cuvette contained *Mt*CS. In this manner, NADH inner-filter effects upon the protein fluorescence could be assessed. The binding of NADH to *Mt*CS causes a quench in protein fluorescence (λ_exc _= 290 nm; 310 ≤ λ_em _≤ 510 nm), and fluorescence values at the maximum emission wavelength (345.5 nm) were plotted against increasing NADH concentrations. The data were fitted to a hyperbolic function, yielding a value of 156 (± 12) μM for the dissociation constant of *Mt*CS-NADH binary complex formation.

### *Mt*CS enzymatic assay for NADH:FMN-oxidoreductase activity

FMN-reductase activity of *Mt*CS was monitored for the forward reaction 25°C in buffer A. The activity measurements were based on the decrease of NADH concentration upon FMN reduction. Owing to the high absorption coefficient of FMN at 360 nm, the consumption of NADH was monitored at 380 nm (ε = 0.893 × 10^3 ^M^-1 ^cm^-1^). The apparent steady state kinetic parameters Km and Vmax of FMN reductase activity were determined for holoenzyme *Mt*CS-FMN_ox _in the presence of varying concentrations of NADH (10, 25, 50, 75, 100, 200, 300 μM). The reaction was started with the addition of 60 nmol of homogeneous *Mt*CS previously incubated with 40 μM FMN_ox_.

### Kinetic isotope effects and proton inventory

[4*S*-^2^H]NADH was synthesized as described by Ottolina *et al*. [51]. The substrates, [4*S*-4-^1^H]- or [4*S*-4-^2^H]-NADH, were purified on a MonoQ column as previously reported [52], and the fractions with absorbance ratios A_260 nm_/A_340 nm _≤ 2.3 were pooled. Both kinetic isotope effects and proton inventory were determined in buffer A with homogeneous *Mt*CS previously incubated with 40 μM FMN_ox_. Primary deuterium kinetic isotope effects were determined from measurements of initial rates in the presence of varying concentrations of either [4*S*-4-^1^H]- or [4*S*-4-^2^H]-NADH, and solvent kinetic isotope effects from measurements of initial rates in the presence of varying concentrations of NADH in either H_2_O or 91 atom % D_2_O. Multiple isotope effects were determined by measuring initial velocities in the presence of varying concentrations of either [4*S*-4-^1^H]- or [4*S*-4-^2^H]-NADH in 90 atom % D_2_O. The pD of buffers used on solvent and multiple isotope effects were measured on a pH meter considering pD = pH + 0.4. Proton inventory was carried out at saturating NADH concentrations at various mole fractions of D_2_O. All measurements were performed in triplicate. The nomenclature proposed by Northrop [31] and Cook and Cleland [34] was used to express isotope effects.

### Data analysis

Values for the apparent kinetic parameters and their standard errors were obtained by fitting the data to the appropriate equations using the non-linear regression function of SigmaPlot 2000 (SPSS, Inc.). The initial rate measured at seven different NADH concentrations were fitted to eq. 1.

(1) v = *V*A/(*K *+ A)

Isotope effect data were fitted to eq 2, which assumes isotope effects on both *V*/*K *and *V*. In equations 1 and 2, *V *is the maximal velocity, *K *is the Michaelis constant, A is substrate concentrations, *E*_*V/K *_and *E*_*V *_are the isotope effects minus 1 on *V*/*K *and *V*, respectively, and *F*_i _is the fraction of deuterium label.

(2) v = *V*A/[*K*(1 + *F*_i_*E*_*V/K*_) + A(1 + *F*_*i*_*E*_*V*_)]

## Abbreviations

CS: chorismate synthase; DAHP: 3-deoxy-D-*arabino*-heptulosonate 7-phosphate; EPSP: 5-enolpyruvylshikimate-3-phosphate; ESI-MS: electrospray ionization-mass spectrometry; FMN: flavin mononucleotide; FMN_ox_: oxidized flavin mononucleotide; FMN_red_: reduced flavin mononucleotide; FPLC: fast protein liquid chromatography; IPTG: isopropyl β-D-thiogalactoside; MDR-TB: multi drug-resistant strains of *M. tuberculosis*; MESG: 2-amino-6-mercapto-7-methylpurine ribonucleoside; *Mt*CS: chorismate synthase from *M. tuberculosis*; *Mt*EPSPS: EPSP synthase from *M. tuberculosis; Mt*SK: shikimate kinase from *M. tuberculosis*; NADH: reduced form of nicotinamide adenine dinucleotide; *Nc*CS: chorismate synthase from *Neurospora crassa*; PCR: Polymerase Chain Reaction; PEP: phosphoenolpyruvate; PNP: purine nucleotide phosphorylase; SDS-PAGE: sodium docecyl sulfate polyacrylamide gel electrophoresis; TB: tuberculosis; XDR-TB: extensively drug-resistant strains of *M. tuberculosis*.

## Authors' contributions

FE carried out protein expression and purification, enzyme kinetic studies, and drafted the manuscript. JESN carried out FMN and NADH binding studies. EKS carried out gene cloning. JF assisted in protein expression and participated in manuscript preparation. MSP carried out mass spectrometry analysis and N-terrminal amino acid sequencing. DSS conceived and coordinated this study, and helped draft the manuscript. LAB participated in experimental design, supervision, and helped draft the manuscript. All authors read and approved the final manuscript.
